# Inhibition of *Escherichia coli* O157:H7 Growth Through Nutrient Competition by Non-O157 *E. coli* Isolated from Cattle

**DOI:** 10.3390/microorganisms13122811

**Published:** 2025-12-10

**Authors:** Joel J. Maki, Kathy T. Mou, Julian Trachsel, Crystal L. Loving

**Affiliations:** 1 Food Safety and Enteric Pathogens Research Unit, National Animal Disease Center, Agricultural Research Service, United States Department of Agriculture, Ames, IA 50010, USA; 2 Agricultural Research Service Participation Program, Oak Ridge Institute for Science and Education, Oak Ridge, TN 37830, USA

**Keywords:** *Escherichia coli*, *Escherichia coli* O157:H7, cattle, commensal, nutrient competition, nutrient exclusion, bacteriocin, probiotics

## Abstract

*Escherichia coli* O157:H7 is a major food safety concern through contamination of beef and produce. Non-antibiotic interventions to minimize O157:H7 in food animals and products are highly desired and one strategy to improve food safety is to reduce O157:H7 in cattle, a main asymptomatic reservoir, through probiotic administration. Non-pathogenic *E. coli* populating the intestine represent a compelling probiotic source, as these strains are already host-adapted. The challenge is to identify non-pathogenic strains capable of competing with O157:H7 for nutrients or those producing compounds to inhibit O157:H7 growth. Here, *E. coli* isolated from cattle fecal and recto-anal junction swab samples were sequenced and screened for the ability to compete with O157:H7. Fourteen genetically distinct, non-Shiga toxin-encoding, non-O157:H7 *E. coli* strains were recovered, and individual isolates representative of each strain were assessed. Nearly all strains possessed complete genetic pathways for the utilization of carbon and nitrogen sources required for O157:H7 colonization of the cattle intestine. Growth curve assays were conducted, and growth metrics were compared between bovine non-O157:H7 *E. coli* isolates and two O157:H7 isolates. While no strain outperformed both O157:H7 strains for all nutrients tested, at least one strain outperformed O157:H7 for each of the carbon sources tested. No strain grew significantly better than O157:H7 in media supplemented with ethanolamine. A “highly competitive” consortium of 4 non-O157:H7 isolates that grew significantly better than O157:H7 reduced O157:H7 counts CFU/mL by 1.53 log_10_ and >0.72 log_10_ under anaerobic and aerobic conditions, respectively, in competition assays. A consortium of “low-competitive” strains reduced O157:H7 counts by >0.47 log_10_ and >0.51 log_10_ under anaerobic and aerobic conditions. These results suggest that cattle harbor non-O157:H7 *E. coli* strains capable of limiting O157:H7 growth in vitro. Surveys of commensal non-O157:H7 isolates from cattle using growth-based phenotypic assays may be useful in identifying *E. coli* strains capable of outcompeting O157:H7 in the bovine intestine for further in vivo testing as probiotics.

## 1. Introduction

Shiga toxin-producing *Escherichia coli* O157:H7 (O157:H7) is an important food safety pathogen, representing >60,000 infections in the US and nearly 3 million infections globally, resulting in >200 deaths annually [1,2,3]. *E. coli* O157:H7 infections are characterized by bloody diarrhea, which has the potential to progress to hemolytic uremic syndrome (HUS) or other life-threatening sequelae, especially in young children and the elderly, with ~50% of cases requiring hospitalization [2]. The high morbidity rate makes finding ways to reduce the entry of O157:H7 into the food supply imperative.

Cattle represent the natural reservoir for O157:H7, which primarily colonizes the recto-anal junction (RAJ) without causing overt clinical disease except in the case of very young calves [4,5]. Post-harvest interventions, such as steam pasteurization and hot water washes, can be effective in reducing O157:H7 contamination of food products, but infections linked to ground beef are still reported yearly. Additional pre-harvest interventions could reduce O157:H7 in cattle prior to processing, further reducing risk of product contamination [6]. Numerous biocontrol strategies have been studied as potential pre-harvest interventions to inhibit O157:H7 colonization and shedding within the bovine host. Biocontrol strategies include vaccination, bacteriophage, and prebiotics, among others, yet none of these methods have been widely adopted by producers [7,8,9].

An O157:H7 reduction method which has recently drawn attention is the concept of competitive exclusion by the commensal microbiota present in the cattle gastrointestinal tract (GIT) [10,11]. Competitive exclusion of organisms, including foodborne pathogens, from the GIT can occur through a variety of mechanisms, including the competition for physical space along the GIT, utilization and/or sequestration of nutrients and ions required by the pathogen for colonization, and production of antimicrobial peptides, such as bacteriocins [12,13]. In theory, strains originating from the cattle GIT should be well-adapted to the environmental conditions therein, providing these isolates increased opportunity to reach and colonize the same environmental niches as O157:H7 [14]. *Escherichia coli* is ubiquitous in livestock species, including cattle [15,16]. Most *E. coli* strains isolated from cattle are harmless commensals, aiding the host in the exclusion of potential pathogens, such as *Salmonella* spp., and playing a role in immune development [17,18]. *E. coli* strains have previously been evaluated and utilized as probiotics, microorganisms capable of conferring health benefits to the host when present in sufficient quantities [19,20]. In fact, *Escherichia coli* Nissle 1917 (Nissle) represents one of the most intensively studied probiotic organisms, with documented anti-inflammatory and pathogen inhibition properties [17,21,22]. Overall, commensal *E. coli* are ideal candidates as potential anti-O157:H7 agents.

Previous research has identified several key nutrients required by O157:H7 for successful colonization of the bovine GIT, including galactose, mannose, ribose, and hexuronates, such as gluconate and glucoronate, as carbon sources, and ethanolamine as a nitrogen source [14,23,24,25,26,27,28]. Previous studies looking at nutrient exclusion of O157:H7 indicate *E. coli* strains, such as *E. coli* Nissle, are capable of outcompeting O157:H7 for select nutrients, preventing colonization of the mouse gut [26]. Thus, nutrient exclusion is a viable strategy that could be applied to prevent, or at least reduce, the colonization and shedding of O157:H7 in cattle.

Bacteriocins are a class of ribosomally encoded and post-translationally modified peptides (RiPPs) produced by bacteria to inhibit the growth of other closely related bacteria within their environment [29,30,31]. *E. coli* are known producers of bacteriocins, with different strains capable of producing both larger (25–80 kDa) colicins and smaller (<10 kDa) microcins. Colicins and microcins function through a variety of mechanisms to inhibit other bacteria within their ecological niche, providing the bacteriocin-encoding organism a competitive advantage, especially when exposed to environmental stressors [30,32]. However, strains encoding a specific bacteriocin are generally resistant to that same bacteriocin, and membrane modifications and modulation of membrane receptors can also confer resistance to the inhibitory effects of bacteriocins [32]. Several studies have assessed the susceptibility of O157:H7 strains to bacteriocins produced by commensal bacteria, with E1, E7, colicin 1b, and colicin V all showing promise as O157:H7 inhibitors both in vitro and in vivo, though activity of bacteriocins can be highly strain-specific [33,34].

To further efforts of identifying non-O157:H7 *E. coli* as a potential probiotic for cattle, we isolated and assessed *E. coli* isolates originating from bovine fecal and RAJ mucosa swabs using in vitro and in silico methods. Of the isolates recovered, we identified 14 unique non-O157:H7 strains and evaluated representative isolates for their ability to inhibit two strains of O157:H7 through nutrient competition and bacteriocin production assays. None of the strains tested produced anti-O157:H7 inhibitory compounds in the supernatant capable of significantly reducing growth of O157:H7. However, several non-O157:H7 *E. coli* isolates displayed enhanced growth characteristics in minimal media supplemented with mannose, ribose, galactose, gluconate, and glucuronate compared to O157:H7. These “highly competitive” strains were combined into a consortium and used in growth competition assays with O157:H7. When combined, the non-O157:H7 consortium significantly reduced O157:H7 outgrowth under both anaerobic and aerobic conditions.

## 2. Materials and Methods

### 2.1. Animals and Sample Collection

Fecal and recto-anal junction mucosa swab samples were collected from calves during a previously published study [35], with samples collected prior to inoculation with *E. coli* O157:H7. All animal protocols were reviewed and approved by the Animal Care and Use Committee at the ARS-National Animal Disease Center in Ames, IA, USA.

### 2.2. Bacterial Strain Isolation and Maintenance

Samples were processed and plated to Sorbitol McConkey Agar (SMAC; BD Difco, Franklin Lakes, NJ, USA) as previously described [35]. Individual colonies were selected, subcultured to SMAC, and presumptively confirmed as non-O157:H7 using latex agglutination (Oxoid, Ogdensburg, NY, USA). Isolates (95 total) were submitted to the National Veterinary Services Laboratories (NVSL; Ames, IA, USA) for taxonomic identification using matrix-assisted laser desorption ionization–time of flight (MALDI-TOF) mass spectrometry (Bruker Biotyper; Bruker Scientific LLC, San Jose, CA, USA). ATCC 700728 and ATCC 43888, two *E. coli* O157:H7 non-Shiga toxin-encoding reference strains, were obtained from the American Type Culture Collection (ATCC; Gaithersburg, MD, USA) and used as O157:H7 indicator strains. All *E. coli* was maintained on tryptic soy agar (TSA; BD Difco) or tryptic soy broth (TSB; BD Bacto) at 39 °C unless otherwise noted to better reflect the temperature of the cattle gastrointestinal tract. For experiments requiring anaerobic atmospheres, a vinyl anaerobic chamber (Coy Laboratories, Grass Lake, MI, USA) was used to maintain anaerobic conditions (5% H_2_: 10% CO_2_: 85% N_2_).

### 2.3. Whole Genome Sequencing and Assembly

Genomic DNA was extracted from the 95 isolates using a DNeasy Blood and Tissue kit following the manufacturer’s instructions (Qiagen, Hilden, Germany). Extracted DNA was quantified, and quality was assessed prior to sequencing on an Illumina HiSEquation (3000) using 150-cycle paired-end reads (Illumina, San Diego, CA, USA). Resultant sequencing reads were trimmed using BBDuk.sh in the BBTools package (v38.90) with the following parameters: ktrim = r k = 23 mink = 11 hdist = 1 tbo tpe minlen = 70 [36]. Trimmed reads were subsampled to ~100× coverage with reformat.sh in BBtools. Read pairs were merged with BBMerge and similar sequences were grouped with Clumpify, both within the BBTools package. The tadpole command within BBtools was used for read error correction before assembly with Spades (v3.15.1) [37]. The BBMap and stats tools within BBTools were used to assess assembly quality. Additional quality metrics were determined using gfastats (v1.3.11) [38].

### 2.4. Genomic Analysis

Genome sequences for *E. coli* ATCC 700728 (Taxon ID 1051353) and *E. coli* ATCC 43888 (Taxon ID 83334) were obtained from the Bacterial and Viral Bioinformatics Resource Center (BV-BRC; https://www.bv-brc.org) accessed on 30 April 2024. Whole genome sequences were analyzed using fastANI (v1.3) through the usegalaxy.eu online platform to assess pairwise average nucleotide identity (ANI) values for each genome compared to all other genomes [39,40]. An ANI value > 99.9% was used to delineate “strain”-level identifications for each isolate [41]. Numbers of contigs, assembly size, and N50 values were used to evaluate assemblies and select a single representative genome for each “strain” if multiple isolates had >99.9% average nucleotide identity. Prokka (v1.14.6) was used to identify coding regions (CDS) and generate .gff files for each genome [42]. Eggnog-mapper (v2.18; database v5.02) was used to provide functional annotations for genomes [43]. A pan-genome of all bovine non-Shiga toxin-producing, non-O157:H7 *E. coli* strains and the two O157:H7 strains used in this study was constructed with Roary (v3.13.0) [44]. The SNP-Sites tool (v2.5.1) was used to identify SNPs and indels between the non-O157:H7 strains using the core genome alignment from Roary [45]. Genome assemblies were analyzed with staramr (v0.9.1) to identify antimicrobial resistance (AMR) genes and plasmids [46]. Plasmids were also identified using MOB-recon within the program MOB-suite (v3.1.9) [47]. Whole genome sequences were assigned O-groups and H-types using ECtyper (v2.0.0) [48]. Abricate (v1.0.1) was used to query the virulence factor database (VFDB) to generate virulence profiles for each isolate [49]. The Bagel5 webserver (http://bagel5.molgenrug.nl; accessed on 27 February 2024) was used to identify bacteriocins and ribosomally synthesized and post-translationally modified peptides (RiPPs) [50]. Biosynthetic gene clusters, including those for bacteriocins, were identified using AntiSmash (v6.1.1) [51]. The kb_DRAM tool (v0.1.2) was used to generate genome-scale metabolic profiles for each assembly [52]. The ggKegg package (v1.1.18) in R (v4.3.1) was used to assess KEGG pathway completeness for specific nutrients of interest [53]. Figure generation was conducted in R using ggplot2 (v3.5.1).

### 2.5. Assessment of Anti-O157 Bacteriocin Production by Cattle Strains

Non-O157:H7 *E. coli* strains were initially assessed for antimicrobial activity against both O157:H7 strains (ATCC 700728 and ATCC 43888) using deferred antagonism assays as previously described [54]. Briefly, non-O157:H7 strains were grown overnight in TSB broth at 39 °C and spotted onto TSA agar plates with and without 0.1 μg/mL mitomycin C (Sigma-Aldrich, Burlington, MA, USA). Plates were incubated overnight at 39 °C to produce individual, well-isolated colonies before being UV-inactivated using a UV Stratalinker 2400 (Stratagene, Santa Clara, CA, USA) set to 3000 kJ × 100 for 30 min. A sloppy agar TSB overlay (0.75% *w*/*v* granulated agar) with 1.0% (*v*/*v*) overnight *E. coli* O157:H7 indicator strain (either ATCC 700728 or ATCC 43888) in TSB was added to the UV-inactivated plate and allowed to set. Plates were incubated overnight at 39 °C after which plates were inspected for zones of inhibition. Each test was conducted in triplicate under both aerobic and anaerobic conditions using both O157:H7 strains. A bacteriocin-producing *E. coli* ATCC 11775 and bacteriocin-sensitive *E. coli* K-12 were used as a positive control to ensure the deferred antagonism assay was functioning as intended.

Non-O157:H7 strains were also assessed for anti-O157:H7 activity using a cell-free supernatant-based growth curve assay using previously described methods [55]. Briefly, bovine non-O157:H7 *E. coli* strains were grown overnight at 39 °C in TSB + 0.1 μg/mL mitomycin C, after which cells were pelleted by centrifugation at 5000× *g* for 20 min. 150 μL of cell-free supernatant was transferred to a 96-well PCR plate and sealed before being placed on the thermocycler and incubating at 95 °C for 20 min to heat-inactivate vegetative cells and bacteriophage present in the cell-free supernatant, enriching for thermostable peptides [56,57]. A TSB + 0.5% (*w*/*v*) agar media was prepared and cooled to 45 °C before addition of an overnight culture of either ATCC 700728 or ATCC 43888 to a final concentration of 0.5% (*v*/*v*). 50 μL of the TSB + 0.5% (*w*/*v*) agar + 0.5% (*v*/*v*) overnight O157:H7 strain was added to each well of a 96-well flat-bottom cell culture plate and allowed to solidify before the addition of 30 μL of heat-inactivated cell-free supernatant. Plates were then sealed and placed onto a plate reader instrument after which the plate was incubated for 24 h at 39 °C and optical density measurements were recorded every 15 min. An Accuris SmartReader 96 MR9600-T instrument (Accuris, Denver, CO, USA) was used to record serial OD_630_ of plates incubated under anaerobic conditions. Cell-free supernatants for all non-O157:H7 *E. coli* strains were tested in quadruplicate using ATCC 700728 and ATCC 43888 as indicator strains in separate experiments. Resultant OD_630_ measurements were imported into R to plot growth curves and area under the logistic curve (AUC) values for each strain with the GrowthCurveR (v0.3.1) package [58]. Statistical analysis (Tukey’s Honestly Significant Difference) and figure generation were conducted with the stats (v3.6.2) and ggplot2 packages. Percent differences in AUC between individual O157:H7 strains grown with heat-inactivated cell-free supernatant (*O157*:*H7 AUC_Treated_*) from individual non-O157:H7 isolates and O157:H7 strains grown in TSB alone (*O157*:*H7 AUC_TSB only_*) was calculated using the following formula:

$$Percent\;difference\;in\;AUC=\;\frac{\left(O157:H7{\;AUC}_{Treated}-O157:H7\;{AUC}_{TSB\;only}\right)}{O157:H7\;{AUC}_{TSB\;only}}\;\times \;100$$

### 2.6. Utilization of Select Carbon and Nitrogen Sources Under Anaerobic Conditions

Ability of *E. coli* strains to utilize carbon and nitrogen sources considered essential for colonization of the anaerobic cattle hindgut: mannose, galactose, ribose, gluconate, glucoronate, and ethanolamine, were assessed with growth curves in minimal media supplemented with individual carbon or nitrogen sources. Briefly, a minimal media composed of 1.0% (*w*/*v*) proteose peptone no. 3 (Difco) + 0.5% *w*/*v* NaCl (Thermo Fisher, Waltham, MA, USA) was supplemented with 1.0% (*w*/*v*) of D-mannose (Sigma), galactose (Sigma), ribose (TCI, Portland, OR, USA), sodium gluconate (AmBeed, Buffalo Grove, IL, USA), or sodium D-glucuronate monohydrate (Thermo Fisher). To assess the ability of strains to utilize ethanolamine as a carbon source, the above minimal media was supplemented with 1.0% (*w*/*v*) ethanolamine hydrochloride (Thermo Fisher) and 150 nM vitamin B12 (TCI) as previously described [59]. To assess ethanolamine as a nitrogen source, media containing of 0.5% (*w*/*v*) NaCl, 0.2% (*w*/*v*) dextrose anhydrous (Thermo Fisher), 150 nM vitamin B12, and 1.0% (*w*/*v*) ethanolamine hydrochloride was prepared as previously described [59].

All *E. coli* strains, including ATCC 700728 and ATCC 43888, were grown in TSB overnight at 39 °C under anaerobic conditions as described above. Cells were pelleted at 5000× *g* for 10 min, and the supernatant was removed. Cells were washed twice with 1× PBS before resuspension in minimal media base w/o additional carbon or nitrogen sources to an OD_600_ of 0.05 (± 0.01). 10 μL of OD_600_-adjusted cell suspension was inoculated into a 96-well cell culture plate containing 190 μL of the single nutrient in minimal media preparations described above. An Accuris SmartReader 96 MR9600-T instrument was used to incubate the plate at 39 °C for 24 h and record serial OD_630_ measurements every 15 min under anaerobic conditions. Each *E. coli* strain was tested in triplicate for each of the carbon and nitrogen sources. Resultant OD_630_ measurements were imported to R to plot growth curves and calculate the mean generation times (T_Gen_; in hours) and mean area under the logistic curve (AUC) values for each isolate with GrowthCurveR. Statistical analysis (Tukey’s Honestly Significant Difference) and figure generation were conducted with the stats and ggplot2 packages. Percent differences in T_Gen_ and AUC between non-O157:H7 *E. coli* strains and individual O157:H7 strains were calculated using the following formulas:

$$Percent\;difference\;in\;T_{Gen}=\;\frac{\left({NonSTEC\;T}_{Gen}-O157:H7\;T_{Gen}\right)}{O157:H7\;T_{Gen}}\;\times \;100$$

$$Percent\;difference\;in\;AUC=\frac{\left(NonSTEC\;AUC-O157:H7\;AUC\right)}{O157:H7\;AUC}\times 100$$

### 2.7. Genome-Wide Association Study (GWAS)

A GWAS analysis was performed to investigate associations between genes and SNPs, and bovine non-O157:H7 *E. coli* strain growth metrics compared to both ATCC 700728 and ATCC 43888 under anaerobic conditions as determined by the GrowthCurveR package (v0.3.1). The gene presence/absence file from Roary and the vcf file from SNP-sites were used to identify gene and SNP-level associations with growth curve characteristics in minimal media supplemented with individual carbon and nitrogen sources using Scoary (v1.6.16) [60]. Associations between SNPs/genes and growth curve characteristics for individual nutrients were considered significant if the Bonferroni-adjusted *p* < 0.05.

### 2.8. In Vitro Competition Assays of Cattle E. coli Isolate Consortia and O157

Non-O157:H7 strains from the minimal media assay were classified as strong and weak utilizers of mannose, galactose, ribose, gluconate, glucuronate, and ethanolamine based on the mean AUC values described above in relation to the mean AUC values of ATCC 700728 and ATCC 43888. Non-O157:H7 strains producing a mean AUC value for a given nutrient that was greater than the mean AUC values for both ATCC 700728 and ATCC 43888 for that same nutrient was considered a strong utilizer of that nutrient, while a strain that produced a mean AUC that was less than the AUC values for one or both of the O157:H7 strains was considered a weak utilizer of that nutrient. Using these data, a high-competitive (HC) consortium of 4 strains, which produced growth curves with significantly greater AUC values than either of the O157:H7 strains for some or all of the carbon and nitrogen sources tested was assembled. A second, low-competitive (LC) consortium of 4 strains which did not produce growth curves with significantly greater AUC values or produced significantly lower AUC values than one or both O157:H7 strains was also assembled. Overnight cultures of all strains in each consortium being tested, as well as ATCC 700728 alone, were grown in the minimal media broth base described above supplemented with 1.0% (*w*/*v*) of mannose, galactose, ribose, gluconate, glucuronate, and ethanolamine hydrochloride (all nutrient media). Strains were incubated overnight at 39 °C under aerobic or anaerobic conditions. Each strain was adjusted to an OD_600_ of 0.05 (± 0.01) and inoculums were diluted 1:100 in all nutrient media. 10 μL of each of the diluted non-O157:H7 strains of a given consortium, as well as 40 μL of diluted ATCC 700728, were inoculated into all nutrient media and incubated for 24 h at 39°C under either aerobic or anaerobic conditions for both the HC and LC consortia. Tubes of all nutrient media inoculated with 40 μL of ATCC 700728 alone were used as a control. Three biological replicates and 2 technical replicates were assessed for each competition group and set of atmospheric conditions. After 24 h, broth cultures were serially log-diluted out to 10^−7^ and dilutions were plated on both SMAC and HardyCHROM O157 chromogenic agar (Hardy Diagnostics, Santa Maria, CA, USA) and plates were incubated aerobically at 39 °C for 24 h. Total CFU/mL counts for ATCC 700728 were determined on both the SMAC and HardyCHROM O157 chromogenic agar plates. Non-O157:H7 colonies were quantified on SMAC only. Colony morphology for all strains was determined on both SMAC and HardyCHROM O157 agar prior to the start of competition assays to confirm the ability to distinguish between O157:H7 and non-O157:H7 isolates on both media types. A selection of sorbitol-negative colonies from SMAC competition plates and mauve colonies from HardyCHROM O157 agar competition plates were confirmed as O157:H7 using an *E. coli* O157 latex agglutination kit (Oxoid). Colony-forming unit (CFU)/mL values were calculated, log_10_-transformed, and statistically compared (*t*-test) in R using the ggpubr (v0.6.0) and dplyr (v1.1.4) packages. All plots were generated with ggplot2 in R.

## 3. Results

### 3.1. Selection of Unique Commensal E. coli Isolates

To identify bovine non-O157:H7 *E. coli* capable of inhibiting *E. coli* O157:H7, *E. coli* isolates were recovered on SMAC from fecal and recto-anal junction mucosa swab specimens from calves. *E. coli* isolates were confirmed as non-O157:H7 if displaying a negative reaction when tested with an O157:H7 latex agglutination kit. A total of 95 confirmed non-O157:H7 isolates were selected for whole genome sequencing (Supplementary Table S1). After sequence assembly, genomes were compared using the FastANI tool to assess isolate relatedness, with isolates sharing >99.9% genomic similarity being considered the same “strain” of *E. coli* (Supplementary Table S2; Supplementary Figure S1). A representative isolate was selected from the different strain clusters based on genome completeness (number of contigs or N50 value), resulting in 21 unique non-O157:H7 *E. coli* strains (Supplementary Table S1), which were then subject to additional bioinformatic analyses.

### 3.2. Genomic Analysis of Unique Non-O157 E. coli Strains

**Figure 1:**
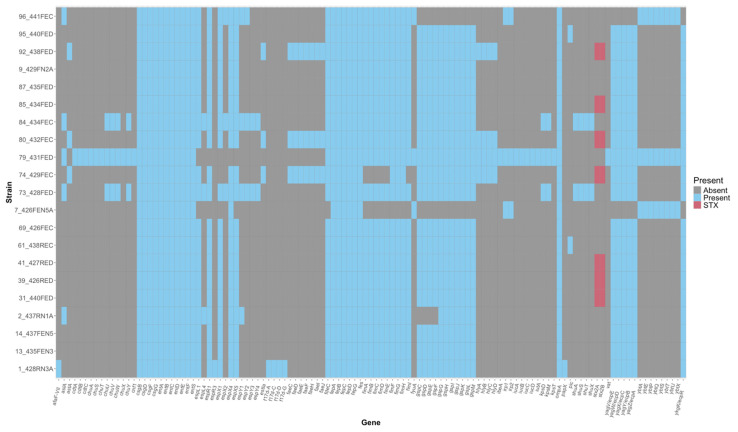
Virulence factors identified in twenty-one genetically distinct bovine non-O157:H7 *Escherichia coli* strains. Virulence factors were identified using Abricate (v1.0.1) to query the virulence factor database (VFDB) and generate virulence profiles for each isolate. Blue cells denote a virulence factor presence while gray cells denote a virulence factor absence. Red cells denote the presence of *stx2A* and *stx2B*, genes required for production of Shiga toxin.

The 21 unique non-O157:H7 *E. coli* genomes were queried against the virulence factor database to assess virulence factor presence (Figure 1). Seven of the 21 unique non-O157:H7 *E. coli* strains harbored Stx2a/b genes and were removed from further analyses, leaving 14 non-Shiga toxin-producing, non-O157:H7 *E. coli* strains for further analysis. Many of the virulence genes identified in the remaining fourteen non-O157:H7 strains are not overtly associated with human disease, though some strains, like 79-431FED, did possess genes that are generally considered to be “true” virulence factors, like *hlyA*, which encodes the hemolysin gene (Figure 1).

**Figure 2:**
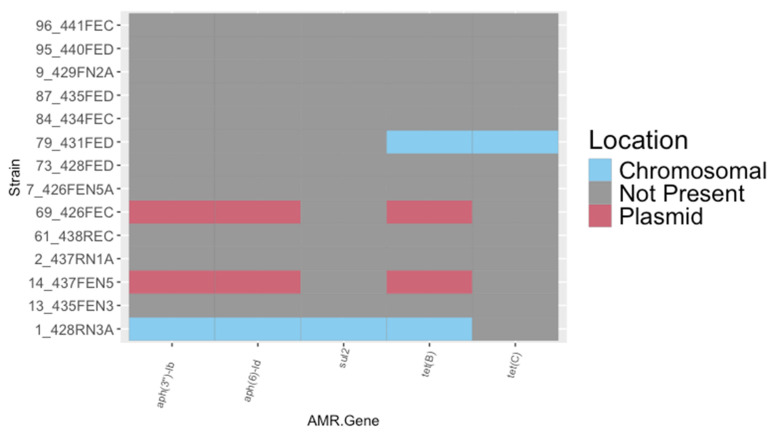
Antimicrobial resistance (AMR) genes identified in the genetically distinct non-O157:H7 strains isolated from cattle. AMR genes were identified using starAMR (v0.9.1). starAMR and MOB-recon (v3.1.9) were used to identify plasmid contigs and assign AMR genes as either chromosomal (blue) or plasmid-associated (red). Plot was generated in ggplot2 (v3.5.1).

The 14 remaining non-O157:H7 strains possessed a variety of AMR genes (Figure 2). Genes associated with resistance to streptomycin (*aph*(*3″*)-*Ib*), kanamycin (*aph*(*6*)-*Id*), sulfamethoxazole (*sul2*), and tetracycline (*tet*(*B*), *tet*(*c*)) were identified, but most of the non-O157:H7 isolates (10/14) did not possess annotated AMR genes in their whole genome assemblies. Isolates 14_437FEN5 and 69_426FEC encoded AMR genes *aph*(*3″*)-*Ib*, *aph*(*6*)-*Id*, and *tet*(*B*). In cross-referencing the sequencing contigs, AMR genes were attributed to plasmid-associated contigs. Isolate 1_428RN3A encoded AMR genes for *aph*(*3″*)-*Ib*, *aph*(*6*)-*Id*, *tet*(*b*), and *sul2*, but none of these AMR genes were found on plasmid-associated contigs. Isolate 79_431FED encoded both *tetB* and *tetC*, and neither of these genes were identified on plasmid-associated contigs (Figure 2). Sequence types and serotypes were determined for each of the 14 non-O157:H7 strains using starAMR and ECTyper (v2.0.0), respectively (Supplementary Table S3).

### 3.3. Phenotypic and Genotypic Assessment of Bacteriocin Production Against O157

**Figure 3:**
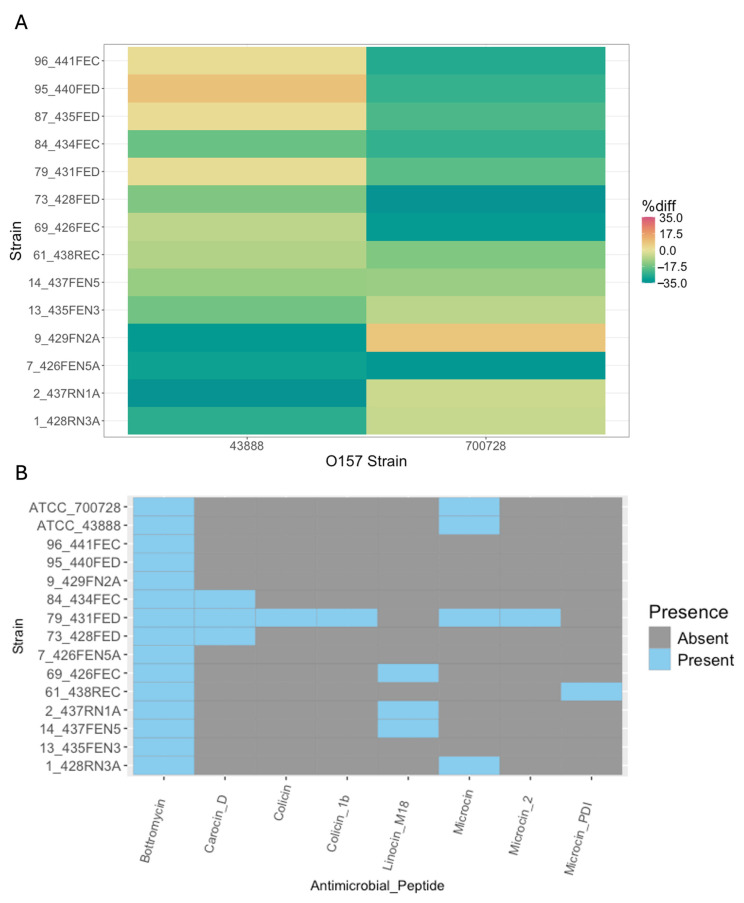
Bacteriocin presence and inhibitory activity of non-O157:H7 *E. coli* strains against O157:H7. (**A**) In vitro inhibition of *E. coli* O157:H7 by heat-inactivated cell-free supernatants of bovine non-O157:H7 strains under anaerobic conditions. AUC values were determined from OD_630_ growth curve data using GrowthCurveR (v0.3.1). Mean AUC values for each of the O157:H7 strains treated with heat-inactivated cell-free supernatant from each of the non-O157:H7 strains were calculated and compared to the mean AUC values for each O157:H7 strain grown in TSB alone to determine the percent difference (%diff) in AUC with dplyr (v1.1.4). Heatmap intensity corresponds to the %diff in the mean area under the curve (AUC) values from growth curves of *E. coli* O157:H7 indicator strains (ATCC 43888 and ATCC 700728) with and without exposure to heat-inactivated, cell-free supernatants of the bovine non-O157:H7 isolates. Four replicates were conducted for each inhibition assay. Statistical comparisons were made between both bovine non-O157:H7 isolate and each of the two *E. coli* O157:H7 strains with tukeyHSD within the stats (v3.6.2) package in R. (**B**) Antimicrobial peptides identified in bovine non-O157:H7 *and E. coli* O157:H7 isolates with Bagel5. Present genes are denoted in blue, and the absence of the gene is denoted in gray.

The 14 non-O157:H7 strains were assessed for their ability to produce anti-O157:H7 antimicrobial compounds, such as bacteriocins. Strains were assessed for in vitro activity against two commercially available strains of *E. coli* O157:H7, ATCC 700728 and ATCC 43888, using both a deferred antagonism assay as well as a growth-based microplate inhibition assay. None of the non-O157:H7 strains produced visible zones of inhibition against either of the O157:H7 strains with the deferred antagonism assay even when 0.1 μg/mL of mitomycin C, an inducer of bacteriocin production, was incorporated into the growth media. Heat-inactivated strain supernatants did not significantly reduce the growth of either O157:H7 strain compared to untreated controls in the quantitative inhibition assay (Figure 3A; Supplementary Table S4).

Individual genes and biosynthetic clusters associated with bacteriocins and other ribosomally synthesized and post-translationally modified peptides (RiPPs) were identified in genomes for the 14 cattle non-O157:H7 strains and the two O157:H7 strains (Figure 3B and Figure S2). Bagel5 annotated more and a greater variety of RiPPs than AntiSmash. Bagel5 identified bottromycin in all the genomes tested, including both O157:H7 genomes. Bagel5 also identified multiple antimicrobial peptides in many of the genomes. Carocin_D was identified in isolates 73_428FED, 79_431FED, and 84_434FEC. Linocin_M18 was identified in isolates 2_437RN1A, 14_437FEN5, and 69_426FEC, while a microcin was identified in isolates 1_428RN3A and 79_431FED, as well as both O157:H7 genomes. Microcin_PDI was identified in isolate 61_438REC. Strain 79_431FED possessed six different classes of antimicrobial peptides, including the previously mentioned bottromycin, carocin_D, and microcin, as well as a colicin, colicin_1b, and microcin_2 (Figure 3B). AntiSmash only identified 4 total RiPP-like genes or gene clusters in the provided genomes. Of these, isolates 14_437FEN5, 69_426FEC, and 79_431FED all possessed genes for colicin V while isolate 2_437RN1A possessed an unidentified RiPP-Like cluster (Supplementary Figure S2).

### 3.4. Phenotypic and Genotypic Nutrient Utilization of O157

The 14 non-O157:H7 strains were assessed for metabolic phenotypes and compared to those of the two O157:H7 strains for nutrient sources previously deemed important for O157:H7 colonization of the bovine GIT: mannose, galactose, ribose, gluconate, glucuronate, and ethanolamine [14,23,24,25,26,27,28]. Growth curves were generated for all strains in response to individual nutrients with shorter generation time (T_Gen_; in hours) and greater area under the growth curve (AUC) values used as a proxy for better growth performance.

To compare strain growth rate in different nutrients, the T_Gen_ of each bovine non-O157:H7 strain was calculated and statistically compared to the T_Gen_ of each of the two O157:H7 strains (Supplementary Figures S3 and S4; Supplementary Table S5). Differences in T_Gen_ between the two O157:H7 strains were observed, with ATCC 43888 generally having longer T_Gen_ for most nutrients compared to ATCC 700728. When comparing T_Gen_ between the 14 bovine non-O157:H7 strains and the two O157:H7 strains, ATCC 43888 T_Gen_ was significantly longer than most strains in minimal media supplemented with galactose or gluconate. While none of the strains had a significantly shorter T_Gen_ than ATCC 700728 in gluconate, strains 61_438REC, 69_426FEC, 84_434FEC, and 87_435FED had significantly shorter T_Gen_ in galactose compared to ATCC 700728, while strains 2_437RN1A and 95_440FED had significantly longer T_Gen_ than ATCC 700728. None of the 14 non-O157:H7 strains had significantly different T_Gen_ than either ATCC 43888 or ATCC 700728 with glucuronate. Strains 7_426FEN5A, 61_438REC, and 87_435FED were the only non-O157:H7 strains that did not have a significantly longer T_Gen_ than ATCC 43888 in mannose. Only strain 95_440FED had a significantly longer T_Gen_ than ATCC 700728 in mannose. In ribose, strain 13_435FEN3 was the only strain to have a significantly shorter T_Gen_ than ATCC 43888, while strains 14_437FEN5, 79_431FED, and 87_435FED displayed significantly longer T_Gen_ compared to ATCC 700728 in minimal media supplemented with ribose (Supplementary Figure S3). Generation times were generated for ethanolamine both as a carbon source and as a nitrogen source (Supplementary Figure S4). Strain 2_437RN1A had a significantly longer T_Gen_ than either of the O157:H7 strains when using ethanolamine as a sole carbon source. There were no other significant differences in T_Gen_ between the two O157:H7 strains and the 14 bovine non-O157:H7 strains when using ethanolamine as either a carbon or nitrogen source (Supplementary Figure S4; Supplementary Table S5). None of the 14 non-O157:H7 strains had a significantly shorter T_Gen_ time than both O157:H7 strains in all nutrients tested.

**Figure 4:**
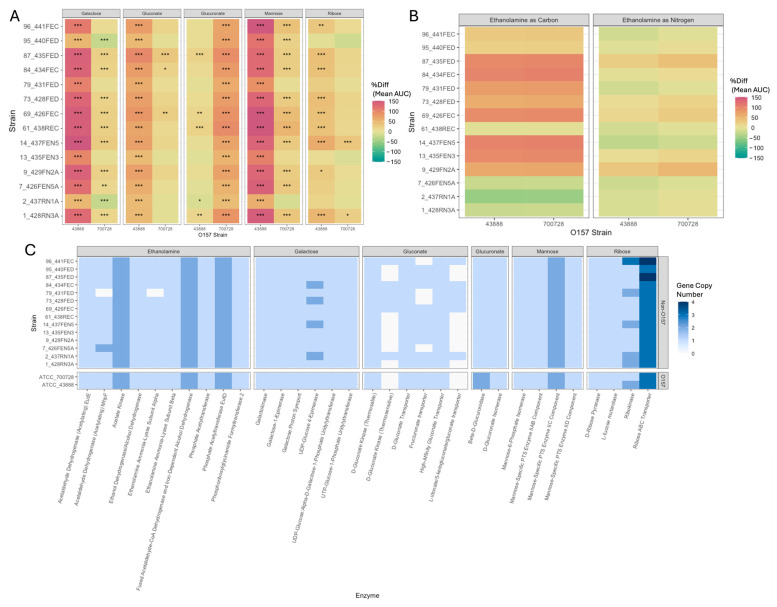
Nutrient utilization profiles of non-O157:H7 *E. coli* strains as compared to two O157:H7 strains. (**A**) Percent difference in mean area under the curve (AUC) values for bovine non-O157:H7 strains compared to two *E. coli* O157:H7 strains (ATCC 43888 and ATCC 700728) in minimal media supplemented with different carbon sources. AUC values were determined from OD_630_ growth curve data using GrowthCurveR (v0.3.1). Mean AUC values for non-O157:H7 strains in each nutrient condition were calculated and compared to the mean AUC values for each O157:H7 strain to determine the percent difference (%diff) in AUC with dplyr (v1.1.4). Heatmap intensity corresponds to the %diff in the mean AUC values. Three replicates were conducted for each strain. Statistical comparisons were made between bovine non-O157:H7 strains and each of the two *E. coli* O157:H7 strains with tukeyHSD within the stats (v3.6.2) package in R. * indicates *p* < 0.05; ** indicates *p* < 0.01; *** indicates *p* < 0.001. (**B**) Percent difference in mean AUC for bovine non-O157:H7 strains compared to ATCC 43888 and ATCC 700728 in minimal media supplemented with ethanolamine as either a carbon or nitrogen source. AUC values were determined from OD_630_ growth curve data using GrowthCurveR. Mean AUC values for non-O157:H7 strains in each nutrient condition were calculated and compared to the mean AUC values for each O157:H7 strain to determine the percent difference (%diff) in AUC with dyplr (v1.1.4). Heatmap intensity corresponds to the %diff in the mean AUC values. Three replicates were conducted for each isolate. Statistical comparisons were made between bovine non-O157:H7 strains and each of the two *E. coli* O157:H7 strains with tukeyHSD using the stats (v3.6.2) package in R. (**C**) KEGG pathway completeness in bovine non-O157:H7 strains and both O157:H7 strains for the catabolism of ethanolamine, galactose, gluconate, glucuronate, mannose, and ribose. Blue denotes a gene as present and the intensity denotes the number of copies of that gene from the Eggnog-mapper (v2.18) annotation of that strain genome. Pathway completeness was determined with ggKegg (v1.1.18).

The logistic AUC was calculated for all strains based on the growth curve data. All 14 of the bovine non-O157:H7 strains tested had a significantly higher AUC compared to ATCC 43888 for galactose, gluconate, and mannose (Figure 4A; Supplementary Table S6). Strains 1_428RN3A, 9_429FN2A, 14_437FEN5, 61_438REC, 69_426FEC, 73_428FED, 84_434FEC, 87_435FED, and 96_441FEC produced significantly greater AUC values than ATCC 43888 in minimal media supplemented with ribose while strains 1_428RN3A, 61_438REC, 69_426FEC, and 87_435FED displayed significantly greater AUCs in glucuronate-supplemented media compared to ATCC 43888. Strain 2_437RN1A had a significantly lower AUC in glucuronate compared to ATCC 43888 (Figure 4A). None of the 14 non-O157:H7 strains tested produced significantly greater AUCs than ATCC 43888 when utilizing ethanolamine as either a carbon or nitrogen source (Figure 4B). All strains produced significantly higher AUCs in glucuronate compared to ATCC 700728. All but strains 2_437RN1A, 13_435FEN3, and 79_431FED produced significantly higher AUCs than 700728 in mannose-supplemented minimal media. Strains 1_428RN3A, 7_426FEN5A, 9_429FN2A, 14_437FEN5, 61_438REC, 69_426FEC, 73_428FED, 84_434FEC, and 87_435FED all produced significantly greater AUC values than ATCC 700728 in galactose-supplemented minimal media while strains 2_437RN1A and 95_440FED had significantly lower AUC values (Figure 4A). Strains 69_426FEC, 84_434FEC, and 87_435FED produced significantly greater AUCs when grown in media with gluconate than ATCC 700728, while strains 1_428RN3A and 14_437FEN5 produced significantly greater AUCs than ATCC 700728 in ribose-containing media. None of the non-O157:H7 strains produced significantly higher AUCs when ethanolamine was present in the media as either a carbon or nitrogen source (Figure 4B; Supplementary Table S6). None of the 14 bovine non-O157:H7 strains produced significantly greater AUC values for all tested nutrients than both *E. coli* O157:H7 strains tested.

Genomic predictions of nutrient utilization patterns for the *E. coli* strains in this study were made using the kb_DRAM (v0.1.2) and ggKegg (v1.1.18) programs (Figure 4C, Figures S5 and S6). DRAM analysis was used to generate a metabolic profile prediction for each of the strains. Few differences were present in genome-encoded metabolic pathways between the bovine non-O157:H7 strains themselves and the two O157:H7 strains. Within the DRAM module “Propionate, pt2”, Propionate CoA-transferase (EC:2.8.3.1) presence varied between the genomes, being identified in 7 of the 14 bovine non-O157:H7 genomes and absent in the two O157:H7 strains. Strain 96_441FEC possessed a GH5 endo-beta-1,4-glucanase/cellulase (EC 3.2.1.4) which was not identified in any of the other genomes, while strain 7_426FEN5A possessed a unique GH43 beta-xylosidase (EC 3.2.1.37) (Supplementary Figure S5). Results from the KEGG module analysis were similar to that of the DRAM analysis, with few differences being observed in module completeness between non-O157:H7 strains and the two O157:H7 strains. Neither O157:H7 strain possessed KEGG modules that were uniquely present or substantially more complete than those in the non-O157:H7 strains. Nearly all non-O157:H7 strains possessed a complete pathway for homoprotocatechuate degradation (KEGG module M00533) which was absent in both O157:H7 strains (Supplementary Figure S6).

A targeted analysis of KEGG pathway completeness for catabolism of galactose, glucuronate, gluconate, mannose, ribose, and ethanolamine were also conducted, revealing subtle differences between strains (Figure 4C). All but strain 79_431FED possessed complete pathways for ethanolamine utilization, with 79_431FED missing one of two acetaldehyde dehydrogenase enzymes (MhpF) and the alpha subunit for the ethanolamine ammonia-lyase enzyme. All bovine non-O157:H7 strains and both O157:H7 strains encoded two gluconate transporters and a thermostable D-gluconate kinase, but several strains also possessed a thermosensitive D-gluconate kinase and additional transporters. All strains possessed the complete metabolic pathways for galactose, glucuronate, mannose, and ribose utilization, however the copy number of individual genes in these pathways did vary between strains (Figure 4C).

A GWAS analysis was conducted with Scoary (v1.6.16) to associate both genes and individual SNPs/indels with significantly greater AUC values compared to both O157:H7 strains when exposed to individual carbon and nitrogen sources in minimal media. None of the Scoary-identified associations were deemed significant after correcting for multiple testing (Bonferroni-adjusted *p* > 0.05).

### 3.5. Consortia of High and Low Competitor Strains vs. E. coli O157

**Figure 5:**
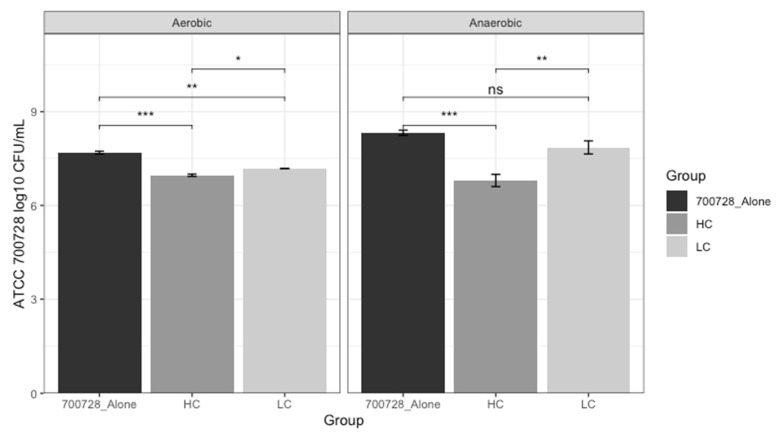
Bar chart of *E. coli* O157:H7 ATCC 700728 counts (log_10_ CFU/mL) in competition assays with high-competitive (HC) and low-competitive (LC) consortia of non-O157:H7 *E. coli* strains. *E. coli* O157:H7 ATCC 700728 counts were assessed after 24 h of co-incubation with HC or LC consortia under aerobic and anaerobic conditions. ATCC 700728 counts were determined on HardyCHROM O157 chromogenic agar plates. Three biological replicates were conducted for each competition assay under each set of conditions. Colony-forming units (CFU)/mL values were calculated, log_10_-transformed, and statistically compared (t.test) in R using the dplyr (v1.1.4) and ggpubr (v0.6.0) packages. * indicates *p* < 0.05; ** indicates *p* < 0.01; *** indicates *p* < 0.001; “ns” indicates *p* > 0.05.

Based on the phenotypic nutrient utilization assays, 2 groups of bovine non-O157:H7 strains were assembled. Strains 14_437FEN5, 61_438REC, 69_426FEC, and 87_435FED produced significantly higher AUC values than both O157:H7 strains (ATCC 700728 and ATCC 43888) for all nutrients tested outside of ethanolamine, and were combined into a consortium which was deemed “highly competitive” (HC) for the nutrients tested. Similarly, strains 2_437RN1A, 13_435FEN3, 73_428FED, and 79_431FED were deemed to be among the poorest utilizers of the nutrients tested based on AUC values and were assembled into a “low-competitive” (LC) consortium. Both consortia were utilized for competition assays against ATCC 700728 and the resulting CFU/mL of ATCC 700728 in the HC, LC, and an unchallenged ATCC 700728 culture were compared to determine the ability of each consortium to reduce O157:H7 growth under both aerobic and anaerobic conditions. ATCC 700728 colony counts were assessed on both HardyCHROM O157 chromogenic agar plates and SMAC agar plates and non-O157:H7 colony counts were determined only on SMAC agar plates (Figure 5, Figures S7 and S8, Supplementary Table S7). Under anaerobic conditions, a significant decrease (*p* < 0.001) in ATCC 700728 after coculture with the HC consortium was observed, resulting in a 1.53 mean log_10_ reduction compared to ATCC 700728 alone. No significant difference was observed in ATCC 700728 levels between the ATCC 700728-alone group and the ATCC 700728 challenged with the LC consortium on the HardyCHROM O157 chromogenic agar, which displayed a mean log_10_ reduction of 0.47 (Figure 5). Results on the SMAC plates under anaerobic conditions were largely similar to those obtained on HardyCHROM, with the HC consortium significantly reducing (*p* < 0.05) ATCC 700728 levels, resulting in a mean log_10_ reduction of 0.85, while LC consortium was unable to significantly reduce ATCC 700728 levels compared to unchallenged controls, showing a mean log_10_ increase of 0.01 (Supplementary Figure S7).

Competition assays were repeated under aerobic conditions to assess impacts of atmospheric conditions on the consortia ability to inhibit ATCC 700728 growth. Both the HC and LC consortia significantly reduced ATCC 700728 levels (HC *p* < 0.001; LC *p* < 0.01) under aerobic conditions, leading to 0.72 log_10_ and 0.51 log_10_ reductions in ATCC 700728 levels, respectively (Figure 5). The results on the HardyCHROM O157 agar plates were mirrored on the SMAC plates, with both the HC and LC groups significantly reducing ATCC 700728 levels by 1.64 log_10_ and 0.85 log_10_, respectively (Supplementary Figure S7; Supplementary Table S7).

Counts of non-ATCC 700728 colonies were assessed on SMAC plates and, under both aerobic and anaerobic conditions, the HC consortium had significantly higher non-ATCC 700728 log_10_ CFU/mL than the LC consortium (Supplementary Figure S8; Supplementary Table S8). The HC consortium displayed 7.97 log_10_ CFU/mL and 8.96 log_10_ CFU/mL under aerobic and anaerobic conditions compared to the LC consortium’s 7.79 log_10_ CFU/mL and 7.00 log_10_ CFU/mL, respectively (Supplementary Figure S8; Supplementary Table S8).

## 4. Discussion

Despite continued efforts to identify effective preharvest interventions for *E. coli* O157:H7 (O157:H7) control in cattle, the pathogen still represents a challenge to the food industry and poses a risk to consumers. Bacteriophage, prebiotics, and vaccines have all been assessed as mechanisms to control O157:H7 colonization and shedding in cattle, resulting in varying levels of success [61]. To date, no single strategy has emerged to eliminate the colonization and shedding of O157:H7 in cattle, suggesting there is a need to further hone existing intervention strategies and combine existing O157:H7 mitigation efforts to enhance efficacy. Probiotic-based interventions represent a key component of a multifaceted approach to control O157:H7 and other Shiga toxin-producing *E. coli* (STEC) in cattle, as the recurrence of STEC detection in beef products highlights the necessity of a multi-hurdle approach involving both pre- and post-harvest interventions. Nutrient competition and bacteriocin production are two important considerations when screening commensal bacteria as probiotic candidates for control of foodborne organisms in the animal host. Here, we sequenced 95 cattle-derived *E. coli* isolates and genetically unique non-O157:H7 strains were queried for bacteriocin production and nutrient utilization to identify isolates capable of inhibiting O157:H7 strains in vitro. All non-O157:H7 *E. coli* strains, as well as two O157:H7 strains (ATCC 700728 and ATCC 43888), were also assessed for growth dynamics in the presence of mannose, galactose, ribose, gluconate, glucuronate, and ethanolamine, nutrients previously determined to be essential for O157:H7 colonization of the cattle intestine [14,23,24,25,26,27]. The goal was to identify cattle-adapted non-O157:H7 strains producing bacteriocins targeting O157:H7 or strains capable of outcompeting O157:H7 for the nutrients it requires to colonize the bovine GIT.

Previous reports have identified bacteriocin-producing strains capable of inhibiting O157:H7 growth, with much of this work focusing on lactic acid bacteria [7,62,63]. Here, we chose to focus on cattle-adapted strains of *E. coli* for several reasons. While *E. coli* is not highly abundant within the cattle intestine, it was hypothesized that non-O157:H7 *E. coli* strains would be more likely to occupy similar metabolic niches within the cattle intestine, making these strains more likely to compete with O157:H7 for essential nutrients in vitro and in vivo [28,64]. Additionally, many bacteriocins have narrow spectra of activity, targeting strains and species that are genetically similar to the producer strain. This is likely due to genetically similar strains occupying similar environments, putting these strains in direct competition for space and resources, necessitating the development of inhibitory compounds such as bacteriocins to provide a competitive advantage [31,65]. These observations made the screening of non-O157:H7 *E. coli* strains a natural choice to identify strains producing anti-O157:H7 compounds and competing with O157:H7 for essential nutrients.

Although 95 *E. coli* isolates were initially isolated for this study, only 21 were unique strains based on their average nucleotide identity scores (>99.9% ANI) [41]. An additional 7 strains were removed from further analysis because they harbored Shiga toxin genes (*stx2A* and *stx2B*). This was not unexpected as over 400 serotypes of STEC have been discovered, with about half of these serotypes implicated in human illness [66,67]. Remaining non-STEC, non-O157:H7 strains were screened for a variety of virulence factors and AMR genes. Many of the strains encoded genes associated with colonization and survival within the host, such as *fimA*, which encodes a type-1 fimbrial subunit identified across a variety of pathogenic and non-pathogenic *E. coli* strains [68,69]. However, some strains, like 79_431FED, did encode “true” virulence factors like *hlyA*, which encodes the hemolysin gene and is associated with human illness [70,71,72,73]. Four of the strains analyzed possessed a mix of AMR genes conferring resistance to streptomycin (*aph(3″)-Ib*), kanamycin (*aph(6)-Id*), sulfamethoxazole (*sul2*), and tetracyclines (*tetB*, *tetC*), with some AMR genes detected on plasmids which have the potential to horizontally transmit to other bacteria [74,75]. While the virulence gene and AMR profiles for some strains may preclude their implementation as potential probiotics, all non-STEC, non-O157:H7 strains were retained for in vitro analyses to provide additional data and hone methods for future, large-scale screening endeavors.

The 14 remaining non-O157:H7 strains were screened for the presence and production of bacteriocins with activity against two *E. coli* O157:H7 strains, ATCC 700728 and ATCC 43888. Assessing potential bacteriocin production against multiple indicator strains of the target of interest is advantageous as different, closely related strains of *E. coli* can have different susceptibility patterns to the same bacteriocin [76]. Genomes were screened for the presence of bacteriocins and other ribosomally encoded and post-translationally modified peptides (RiPPs) using 2 different tools, Bagel5 and antiSMASH [50,51]. The tools generated different bacteriocin profiles for the isolates, highlighting the difficulty in making accurate RiPP-predictions based on genomics alone. Previous comparisons of RiPP-mining tools have noted similar discrepancies, suggesting the utilization of multiple tools to add power to the identification of bacteriocins in genomic data [77,78]. The different RiPP-mining tools did identify genes for colicin V and colicin Ib, which can inhibit certain strains of O157:H7 [33,34,79]. Despite the utilization of multiple indicator strains (ATCC 700728 and ATCC 43888), and inclusion of a well-established bacteriocin inducer (mitomycin C) in screening media preparations, none of the non-O157:H7 strains produced bacteriocins capable of inhibiting either O157:H7 strain in vitro. This lack of inhibition could be due to the fact that many strains of potential bacteriocin-producing bacteria display diminished or inconsistent production of bacteriocins when removed from their natural environment [54,80]. Additionally, *E. coli* strains are frequently resistant to the effects of colicins and microcins [31,32,81,82]. A similar study of *E. coli* from cattle found ~1% of isolates were capable of producing inhibitory colicins and the majority of these colicin-producing strains were *E. coli* O157:H7 [83]. Together, these results highlight the need to screen larger libraries of diverse bacterial isolates using a variety of conditions against multiple indicator strains to ensure maximal identification of inhibitory strains and compounds [82,84,85,86].

Annotations of metabolism related genes and pathways highlighted similarities in metabolic potential across *E. coli* strains. All strains possessed complete genetic pathways for the utilization of galactose, gluconate, mannose, and gluconate, and nearly all strains possessed the genes necessary for ethanolamine utilization, suggesting nearly all strains could grow in the presence of all nutrients tested. This was not surprising as these strains originated from the cattle GIT, and bacterial strains originating from similar environments tend to encode similar metabolic genes and pathways [87,88]. Despite the overall similarity, slight differences were identified between individual strains, and between the O157:H7 and non-O157:H7 strains both at the gene and pathway levels. One difference of note was the presence of a complete pathway for homoprotocatechuate degradation which was exclusively identified in non-O157:H7 strains and completely absent in both O157:H7 genomes. The homoprotocatechuate degradation pathway is responsible for the degradation of hydroxyphenylacetic acid, a product of tyrosine, proanthocyanidin, and flavonoid degradation, all of which are compounds found in the cattle GIT [89,90]. This differentially abundant catabolic pathway could be leveraged for the development of a potential synbiotic or feed additive to enhance the outgrowth of non-O157:H7 *E. coli*, aiding in competitive exclusion of O157:H7 and other STEC strains.

Strains were assessed for the ability to grow in minimal media supplemented with different carbon and nitrogen sources known to be required for O157:H7 colonization of the cattle GIT [14,23,24,25,26,27,28]. In vitro growth curve assays for individual nutrients in minimal media were conducted under anaerobic conditions for the cattle-derived non-O157:H7 strains, as well as the two O157:H7 indicator strains and growth performance was assessed by comparing generation times and AUC values. All growth curve assays were conducted under anaerobic conditions to better simulate conditions in the cattle hindgut [91,92,93]. Most non-O157:H7 strains grew significantly better than both O157:H7 strains in minimal media supplemented with galactose and mannose. For gluconate and ribose, most non-O157:H7 strains produced greater AUCs than ATCC 43888, but few generated significantly greater AUC values than ATCC 700728. All non-O157:H7 isolates produced significantly greater AUC values in glucuronate than ATCC 70728, but only four had significantly greater AUC values than ATCC 43888. None of the non-O157:H7 *E. coli* strains produced greater AUC values in minimal media supplemented with ethanolamine as either a carbon or nitrogen source. Growth patterns for ATCC 700728 and ATCC 43888 were different for all nutrients tested, suggesting underappreciated metabolic diversity within O157:H7. The in vitro nutrient utilization profiling here suggests further screens to identify cattle-derived non-O157:H7 strains capable of nutrient competition against *E. coli* O157:H7 should focus on gluconate, glucoronate, ribose, and ethanolamine. Of these, ethanolamine is particularly important as it is utilized by other potential food safety-relevant pathogens, like *Salmonella*, making identification of efficient ethanolamine utilizers a high priority [59,94,95]. Some strains displayed shorter generation times than others but generated smaller AUC values, suggesting that, under a given condition, a strain grew more quickly but reached a lower terminal cell density (OD_630_). This suggests that different strains of *E. coli* display different maximal carrying capacities in different broth culture compositions, likely due to intraspecies competition and cooperation dynamics that vary between strains and growth conditions [96,97]. Another key finding of this work was that no single bovine-derived non-O157:H7 strain was capable of growing significantly better than both O157:H7 strains for all nutrients tested, highlighting the limitations of single strain probiotic interventions when implemented for the purpose of nutrient exclusion, a finding confirmed by others [18,26,98].

Despite having similar genetic profiles, there were differences in metabolic phenotypes between the bovine non-O157:H7 strains and O157:H7. A genome-wide association study (GWAS) analysis did not identify any specific genes, single nucleotide polymorphisms (SNPs), or insertions/deletions (indels) within the core genome that explained which strains would significantly outperform the O157:H7 strains for specific nutrients, though this could have been an issue with the small sample size reducing statistical power [99]. Others have noted that differences in expression level and/or DNA methylation can be used to explain differences in metabolic phenotype between strains possessing similar metabolic gene repertoires [88,100]. Overall, no distinct genetic signature was identified to differentiate non-O157:H7 *E. coli* strains capable of outgrowing O157:H7 for the individual nutrients in this study. Further work incorporating additional *E. coli* strains (including additional O157:H7 strains) could help identify genetic signatures associated with the ability to outperform O157:H7 for individual nutrients and allow for more rapid screening of *E. coli* isolates as potential probiotic candidates, though the results presented here suggest in vitro growth assays are still required to confirm growth characteristics.

As no single strain grew significantly better than both ATCC 700728 and ATCC 43888 for all nutrients tested, two different consortia of non-O157:H7 strains were generated based on in vitro nutrient utilization profiles compared to O157:H7, one “highly competitive” (HC) consortium and one “low-competitive” (LC) consortium. ATCC 700728 grew to greater AUC values for all nutrients outside of mannose, so it was selected as the O157:H7 strain used for competition against the two consortia. Under anaerobic conditions, the HC consortium significantly reduced O157:H7 growth while the LC consortium did not, suggesting growth performance, as determined by AUC values from growth curves on specific substrates, can be used to predict the ability to nutritionally outcompete bacterial strains of interest. To assess the generalizability of the assembled consortia in different environments, the competition assays were repeated under aerobic conditions. Under aerobic conditions, the inhibitory effects of the HC consortium on ATCC 700728 growth were still significant, though less pronounced. These results highlight the importance of understanding the environmental context in which a probiotic or probiotic consortium will be utilized and ensuring probiotic strains are evaluated under relevant environmental conditions, a finding in line with other studies [101,102]. The LC consortium also resulted in a significant reduction in O157:H7 growth under aerobic conditions. While this reduction was significant, the growth inhibition of ATCC 700728 by the LC consortium was still significantly less than the reduction observed with the HC consortium, suggesting that even outside of the initial screening conditions, high performing strains may still display benefits to target inhibition compared to other, lower-performing strains. However, this association will need to be confirmed through testing and inclusion of additional *E. coli* strains in various combinations to optimize selection of candidate strains for incorporation into future anti-O157:H7 consortia.

One limitation of the current study is that, while two indicator strains of *E. coli* O157:H7 were utilized for both the bacteriocin production and nutrient growth curve assays, neither ATCC 700728 nor ATCC 43888 produce Shiga toxin. This choice was intentional, as it reduced risk to technical staff and reduced personal protective equipment requirements. However, it is thought that Shiga toxin and the phages that carry it may aid in elimination of bacterial competitors, conferring a competitive advantage to STEC and other Shiga toxin-producing bacteria [103]. Follow-up studies should incorporate both non-*stx* and *stx*-encoding O157:H7 isolates to ensure results are applicable to a broad range of O157:H7 strains, and other foodborne serotypes of STEC.

Together, the results of this work highlight the importance of phenotypic screening under relevant environmental conditions when assessing bacterial strains as potential probiotics against O157:H7, a finding noted by others [104,105]. Our incorporation of two indicator strains into the in vitro assays ensured the results of these assays were not strain-specific [54,106]. Future work could broaden the number of indicator strains used to better control for intrastrain variability present within the O157:H7 serotype. Additional studies could broaden to target other highly abundant nutrients present in the cattle intestine that may impact O157:H7 colonization success, such as malate and fumarate [107,108]. Overall, this work developed a viable strategy for the sensible screening of bacteria for probiotic properties and their assembly into consortia capable of competing nutritionally with *E. coli* O157:H7. The concepts and assays described herein could be applied to a multitude of pathogens and other target organisms. Future studies will need to be conducted to determine the effectiveness of similar consortia of bovine *E. coli* against additional O157:H7 strains both in vitro and in vivo.

## 5. Conclusions

Here, we isolated fourteen genetically distinct *E. coli* from bovine fecal and recto-anal junction samples and evaluated their ability to inhibit *E. coli* O157:H7 through antimicrobial peptide production and nutrient exclusion. While none of the non-O157:H7 strains produced anti-O157:H7 inhibitory compounds, several of the strains displayed enhanced growth characteristics in media supplemented with mannose, ribose, galactose, gluconate, and glucuronate compared to O157:H7. These “highly competitive” strains were combined into a consortium and used in competition assays with O157:H7, significantly reducing O157:H7 growth under anaerobic and aerobic conditions. A second consortium of lower-performing strains was also constructed and competed against O157:H7, resulting in significantly less O157:H7 growth inhibition compared to the highly competitive group. Further work will need to evaluate the effectiveness of similarly constructed consortia against additional O157:H7 strains both in vitro and in vivo.

## Data Availability

The whole genome sequences of the 95 bovine *E. coli* strains have been deposited at GenBank under bioproject PRJNA1309676 (https://www.ncbi.nlm.nih.gov/bioproject/?term=PRJNA1309676, 22 August 2025).

## Supplementary Materials

The following supporting information can be downloaded at https://www.mdpi.com/article/10.3390/microorganisms13122811/s1, Figure S1. Heatmap of pairwise average nucleotide identity (ANI) values for bovine non-O157:H7 *Escherichia coli* isolates; Figure S2. Heatmap of secondary metabolite biosynthesis gene clusters identified in unique bovine non-O157:H7 *E. coli* strains and two *E. coli* O157:H7 (ATCC 43888 and ATCC 700728) strains with AntiSmash (v6.1.1); Figure S3. Heatmap of the percent difference in the mean generation time (T_Gen_) for unique bovine non-O157:H7 *E. coli* strains compared to two *E. coli* O157 strains (ATCC 43888 and ATCC 700728) when grown in minimal media supplemented with indicated carbon source in anaerobic conditions; Figure S4. Heatmap of the percent difference in the mean generation time (T_Gen_) for unique bovine non-O157:H7 *E. coli* strains compared to two *E. coli* O157:H7 strains (ATCC 43888 and ATCC 700728) when grown in minimal media supplemented with ethanolamine as either a carbon or nitrogen source; Figure S5. Heatmap of DRAM metabolic annotations of unique bovine non-O157:H7 *E. coli* strains and O157:H7 strains (ATCC 43888 and ATCC 700728); Figure S6. Heatmap of KEGG module completeness in unique bovine non-O157:H7 *E. coli* strains and O157:H7 strains (ATCC 43888 and ATCC 700728); Figure S7. Barcharts of *E. coli* O157:H7 ATCC 700728 counts (log_10_ CFU/mL) in competition assays with high-competitive (HC) and low-competitive (LC) consortia of unique bovine non-O157:H7 *E. coli* strains; Figure S8. Barcharts of bovine non-O157:H7 *E. coli* strain counts (log_10_ CFU/mL) in competition assays of high-competitive (HC) and low-competitive (LC) consortia of unique bovine non-O157:H7 strains against *E. coli* O157:H7 ATCC 700728; Table S1. Bovine *E. coli* isolate metadata and assembly statistics; Table S2. Pairwise average nucleotide identity (ANI) values for bovine *E. coli* isolate assemblies. ANI values were determined with FastANI (v1.3); Table S3. Sequence types and serotypes for genetically distinct unique bovine non-O157:H7 *E. coli* strains; Table S4. Area under the curve (AUC) values of *E. coli* O157:H7 strains (ATCC 43888 and ATCC 700728) exposed to heat-inactivated, cell-free supernatants from unique bovine non-O157:H7 *E. coli* strains; Table S5. Generation times (T_Gen_) values of unique bovine non-O157:H7 *E. coli* strains and *E. coli* O157:H7 strains (ATCC 43888 and ATCC 700728) in minimal media supplemented with ethanolamine, galactose, gluconate, glucuronate, mannose, and ribose; Table S6. Area under the curve (AUC) values of unique bovine non-O157:H7 *E. coli* strains and *E. coli* O157:H7 strains (ATCC 43888 and ATCC 700728) in minimal media supplemented with ethanolamine, galactose, gluconate, glucuronate, mannose, and ribose; Table S7. *E. coli* O157:H7 ATCC 700728 counts (log_10_ CFU/mL) in competition assays with high-competitive (HC) and low-competitive (LC) consortia of unique bovine non-O157:H7 *E. coli* strains; Table S8. Bovine non-STEC isolate counts (log_10_ CFU/mL) in competition assays with high-competitive (HC) and low-competitive (LC) consortia of unique bovine non-O157:H7 *E. coli* strains against *E. coli* O157:H7 ATCC 700728.
