# Supplementary material for: Inhibition of Escherichia coli O157:H7 Growth Through Nutrient Competition by Non-O157 E. coli Isolated from Cattle
Source: Microorganisms. 2025 Dec 10;13(12):2811. doi: 10.3390/microorganisms13122811 (PMC12735539; doi:10.3390/microorganisms13122811)
Supplement: Supplementary file 1 [file microorganisms-13-02811-s001.zip › Supplementary File 1_v4.pdf]

Supplementary File 1: Supplementary Figures and Supplementary Table Legends

Inhibition of *Escherichia coli* O157:H7 Growth Through Nutrient Competition by non-O157 *E. coli* isolated from cattle

Authors: Joel J Maki[1], Kathy T Mou[1,2], Julian Trachsel[1], and Crystal L Loving[1]

Table of Contents:

Supplementary Figure 1. Heatmap of pairwise average nucleotide identity (ANI) values for bovine non-O157:H7 *Escherichia coli* isolates.

Supplementary Figure 2. Heatmap of secondary metabolite biosynthesis gene clusters identified in unique bovine non-O157:H7 *E. coli* strains and two *E. coli* O157:H7 (ATCC 43888 and ATCC 700728) strains with AntiSmash (v6.1.1).

Supplementary Figure 3. Heatmap of the percent difference in the mean generation time (T_{Gen}) for unique bovine non-O157:H7 *E. coli* strains compared to two *E. coli* O157 strains (ATCC 43888 and ATCC 700728) when grown in minimal media supplemented with indicated carbon source in anaerobic conditions.

Supplementary Figure 4. Heatmap of the percent difference in the mean generation time (T_{Gen}) for unique bovine non-O157:H7 *E. coli* strains compared to two *E. coli* O157:H7 strains (ATCC 43888 and ATCC 700728) when grown in minimal media supplemented with ethanolamine as either a carbon or nitrogen source.

Supplementary Figure 5. Heatmap of DRAM metabolic annotations of unique bovine non-O157:H7 *E. coli* strains and O157:H7 strains (ATCC 43888 and ATCC 700728).

Supplementary Figure 6. Heatmap of KEGG module completeness in unique bovine non-O157:H7 *E. coli* strains and O157:H7 strains (ATCC 43888 and ATCC 700728).

Supplementary Figure 7. Barcharts of *E. coli* O157:H7 ATCC 700728 counts (log_{10} CFU/mL) in competition assays with high competitive (HC) and low competitive (LC) consortia of unique bovine non-O157:H7 *E. coli* strains.

Supplementary Figure 8. Barcharts of bovine non-O157:H7 *E. coli* strain counts (log_{10} CFU/mL) in competition assays of high competitive (HC) and low competitive (LC) consortia of unique bovine non-O157:H7 strains against *E. coli* O157:H7 ATCC 700728.

Supplementary Table 1. Bovine *E. coli* isolate metadata and assembly statistics. Assembly statistics were determined using gfastats (v1.3.11). Isolates were grouped into an "ANI cluster" based on the results from FastANI (v1.3). Isolates sharing >99.9% average nucleotide identity (ANI) were placed in the same cluster (denoted with letters). Isolates that do not have a letter (N/A) shared <99.9% ANI with all other isolates. One isolate from each cluster and unique isolates that were selected for further *in silico* and *in vitro* analyses are denoted. An "X" signifies an isolate was non-Shiga toxin-encoding while an "O" signifies the isolate as Shiga toxin-encoding and was removed prior to downstream analyses.

Supplementary Table 2. Pairwise average nucleotide identity (ANI) values for bovine *E. coli* isolate assemblies. ANI values were determined with FastANI (v1.3).

Supplementary Table 3. Sequence types and serotypes for genetically distinct unique bovine non-O157:H7 *E. coli* strains. Sequence types were determined with starAMR (v0.9.1) and serotypes were determined with ECtyper (v2.0.0).

Supplementary Table 4. Area under the curve (AUC) values of *E. coli* O157:H7 strains (ATCC 43888 and ATCC 700728) exposed to heat-inactivated, cell-free supernatants from unique bovine non-O157:H7 *E. coli* strains. AUC values were determined from OD_{630} growth curve data using GrowthCurveR (v0.3.1). The mean AUC values and standard deviations for each of the O157:H7 strains treated with heat-inactivated cell-free supernatant from each of the unique non-O157:H7 strains and a TSB-only control were calculated with dplyr (v1.1.4).

Supplementary Table 5. Generation times (T_{Gen}) values of unique bovine non-O157:H7 *E. coli* strains and *E. coli* O157:H7 strains (ATCC 43888 and ATCC 700728) in minimal media supplemented with ethanolamine, galactose, gluconate, glucuronate, mannose, and ribose. Ethanolamine was provided as both a carbon (C) and nitrogen (N) source. T_{Gen} values were determined from OD_{630} growth curve data using GrowthCurveR (v0.3.1). The mean T_{Gen} values and standard deviations were calculated with dplyr (v1.1.4).

Supplementary Table 6. Area under the curve (AUC) values of unique bovine non-O157:H7 *E. coli* strains and *E. coli* O157:H7 strains (ATCC 43888 and ATCC 700728) in minimal media supplemented with ethanolamine, galactose, gluconate, glucuronate, mannose, and ribose. Ethanolamine was provided as both a carbon (C) and nitrogen (N) source. AUC values were determined from OD_{630} growth curve data using GrowthCurveR (v0.3.1). The mean AUC values and standard deviations were calculated with dplyr (v1.1.4).

Supplementary Table 7. *E. coli* O157:H7 ATCC 700728 counts (log_{10} CFU/mL) in competition assays with high competitive (HC) and low competitive (LC) consortia of unique bovine non-O157:H7 *E. coli* strains. *E. coli* O157:H7 ATCC 700728 counts were assessed after 24h of co-incubation with HC or LC consortia at 39°C under either aerobic or anaerobic conditions. Counts were determined on both HardyCHROM O157 agar plates and Sorbitol MacConkey agar plates. Mean and standard deviation values were calculated with dplyr (v1.1.4).

Supplementary Table 8. Bovine non-STEC isolate counts (log_{10} CFU/mL) in competition assays with high competitive (HC) and low competitive (LC) consortia of unique bovine non-O157:H7 *E. coli* strains against *E. coli* O157:H7 ATCC 700728. Bovine non-O157:H7 *E. coli* strain counts were assessed after 24h of co-incubation with *E. coli* O157:H7 ATCC 700728 at 39°C under either aerobic or anaerobic conditions. Counts were determined on Sorbitol MacConkey agar plates. Mean and standard deviation values were calculated with dplyr (v1.1.4).

Supplementary Figures:

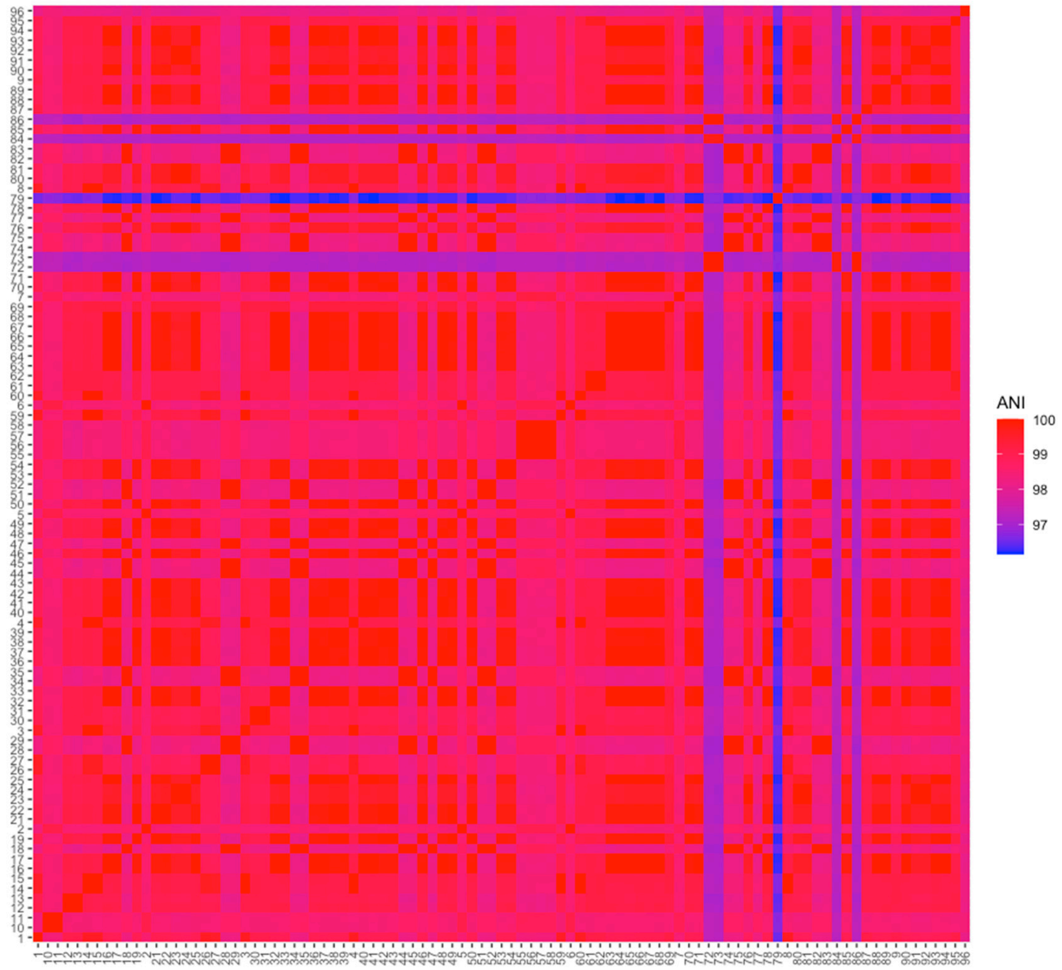

Supplementary Figure 1. Heatmap of pairwise average nucleotide identity (ANI) values for bovine non-O157:H7 *Escherichia coli* isolates. ANI values were determined with FastANI (v1.3) and plot was made in R (v4.3.1) using ggplot2 (v3.5.1).

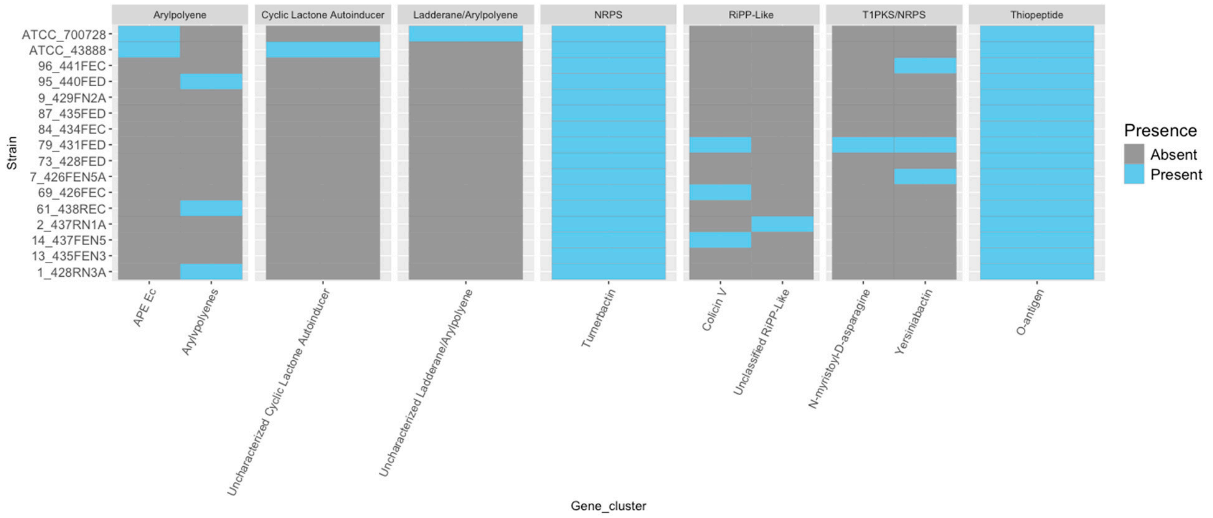

Supplementary Figure 2. Heatmap of secondary metabolite biosynthesis gene clusters identified in unique bovine non-O157:H7 *E. coli* strains and two *E. coli* O157:H7 (ATCC 43888 and ATCC 700728) strains with AntiSmash (v6.1.1). Present gene clusters are denoted in blue, and the absence of the gene is denoted in gray.

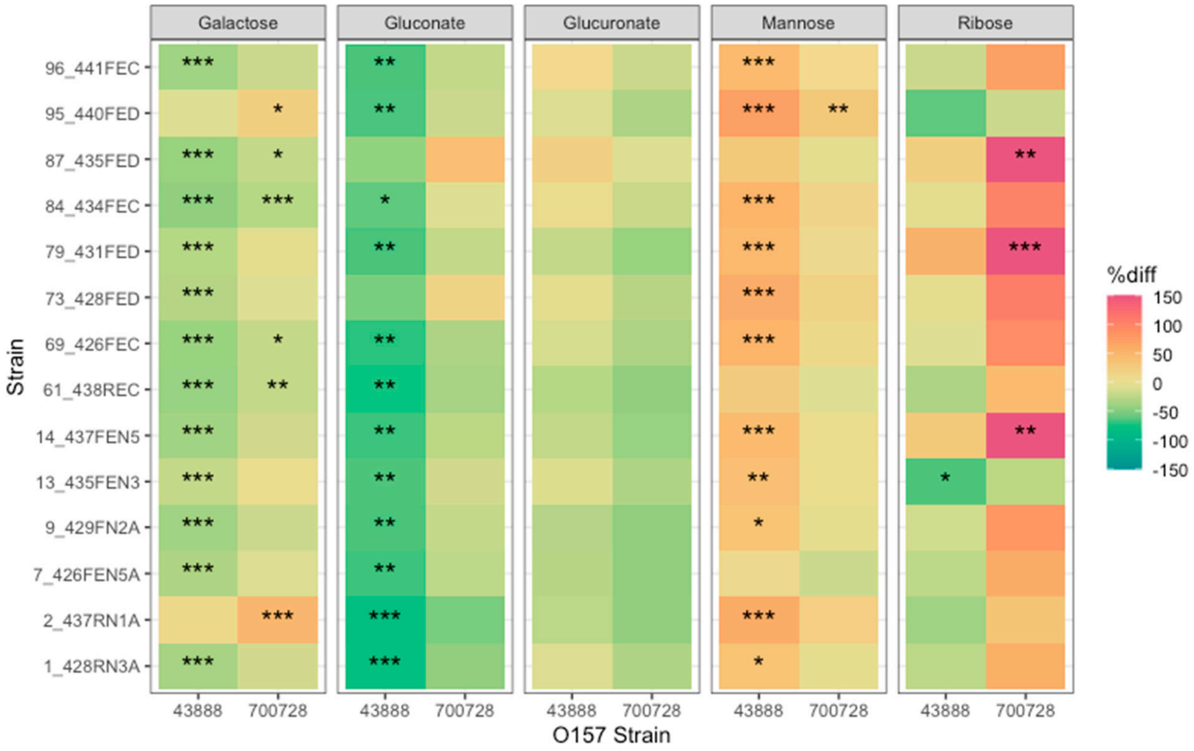

Supplementary Figure 3. Heatmap of the percent difference in the mean generation time (T_{Gen}) for unique bovine non-O157:H7 *E. coli* strains compared to two *E. coli* O157 strains (ATCC 43888 and ATCC 700728) when grown in minimal media supplemented with indicated carbon source in anaerobic conditions. AUC values were determined from OD_{630} growth curve data using GrowthCurveR (v0.3.1). Three replicates were conducted for each isolate. The mean T_{gen} values for each non-O157:H7 *E. coli* strain was calculated and compared to the mean T_{Gen} values for each O157:H7 strain to determine the percent difference in T_{Gen} (%diff). A %diff > 0 indicates a strain had a longer T_{Gen} than the O157 strain it is being compared to while a %diff < 0 indicates a strain had a shorter T_{Gen} than the O157 strain it is being compared to. Statistical comparisons were made between the mean T_{Gen} values of non-O157:H7 strain and each of the two *E. coli* O157:H7 strains with tukeyHSD using the stats (v3.6.2) package in R. * indicates $p < 0.05$; ** indicates $p < 0.01$; *** indicates $p < 0.001$.

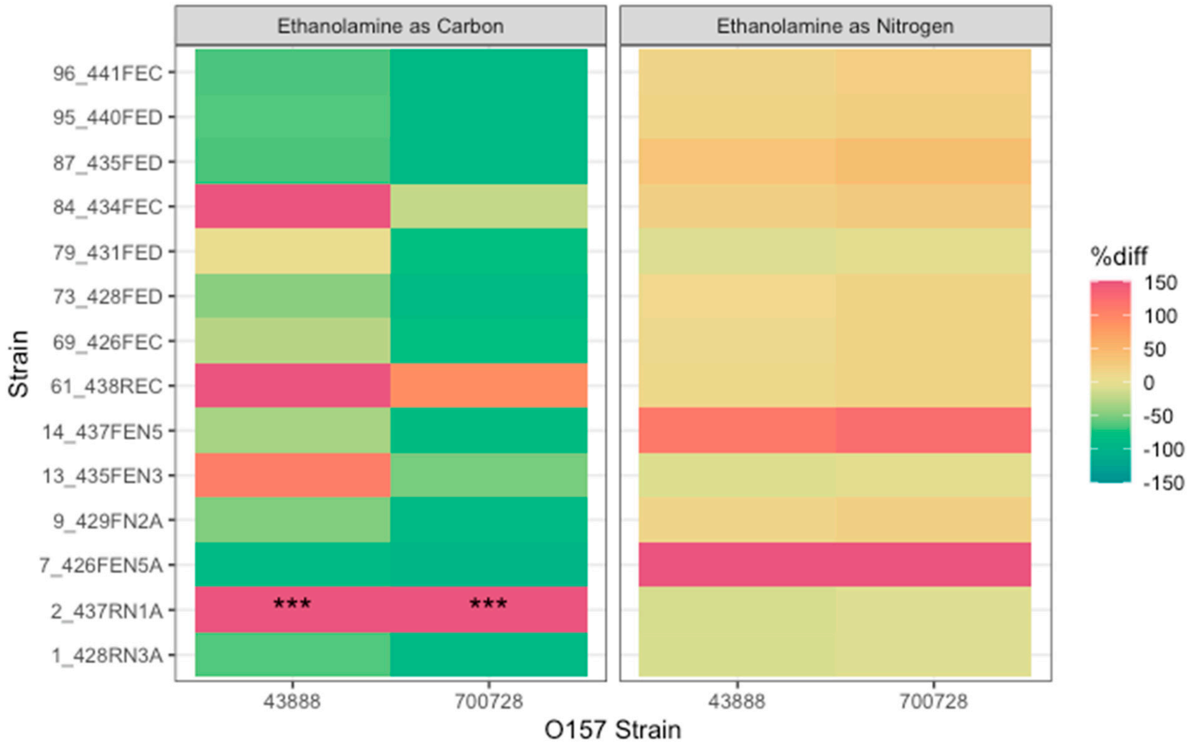

Supplementary Figure 4. Heatmap of the percent difference in the mean generation time (T_{Gen}) for unique bovine non-O157:H7 *E. coli* strains compared to two *E. coli* O157:H7 strains (ATCC 43888 and ATCC 700728) when grown in minimal media supplemented with ethanolamine as either a carbon or nitrogen source. AUC values were determined from OD_{630} growth curve data using GrowthCurveR (v0.3.1). Three replicates were conducted for each isolate. The mean T_{gen} values for each non-O157:H7 strain was calculated and compared to the mean T_{Gen} values for each O157:H7 strain to determine the percent difference in T_{Gen} (%diff). A %diff > 0 indicates a strain had a longer T_{Gen} than the O157 strain it is being compared to while a %diff < 0 indicates a strain had a shorter T_{Gen} than the O157 strain it is being compared to. Statistical comparisons were made between the mean T_{Gen} values of non-O157:H7 strain and each of the two *E. coli* O157:H7 strains with tukeyHSD using the stats (v3.6.2) package in R. * indicates $p < 0.05$; ** indicates $p < 0.01$; *** indicates $p < 0.001$.

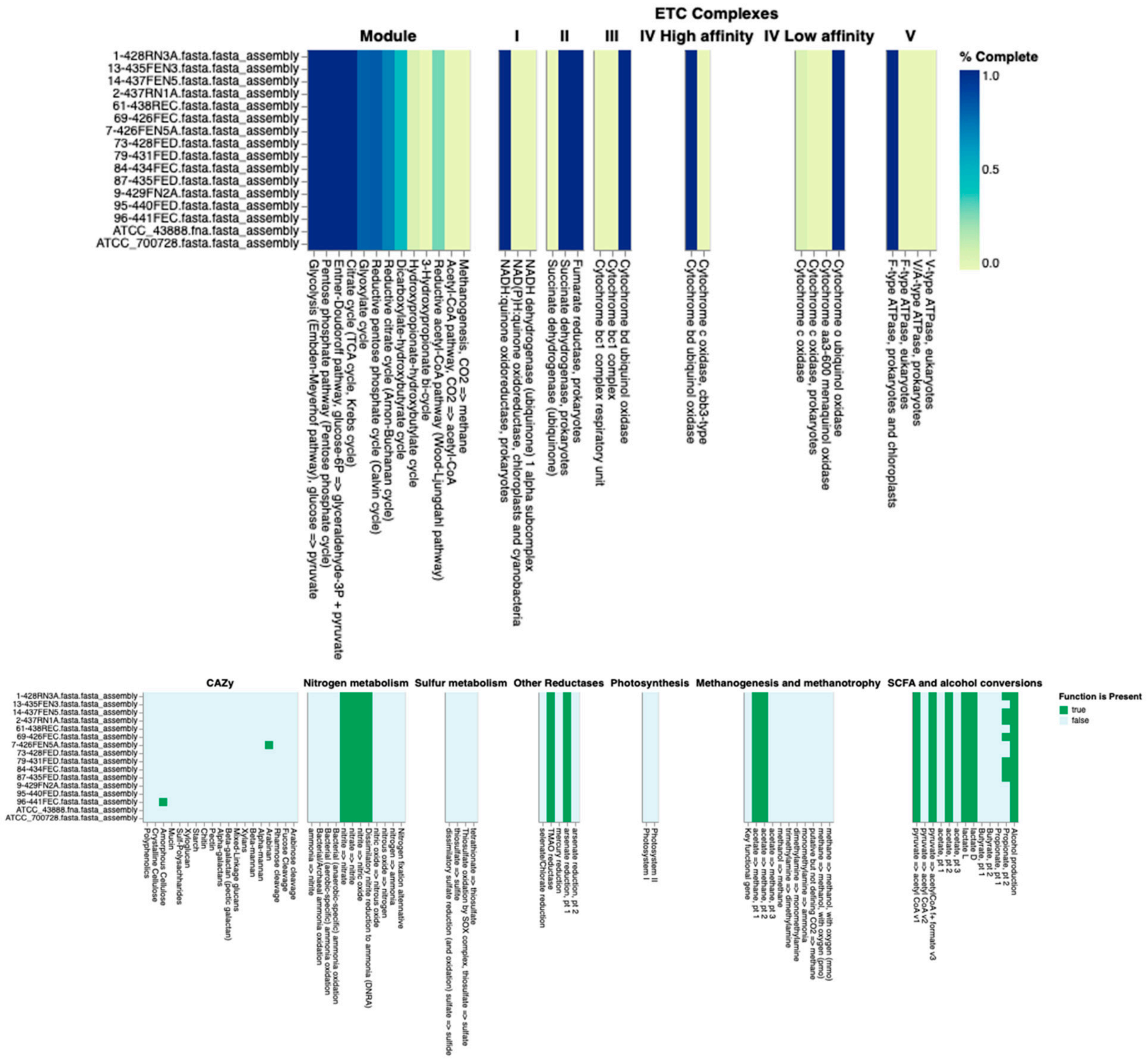

Supplementary Figure 5. Heatmap of DRAM metabolic annotations of unique bovine non-O157:H7 *E. coli* strains and O157:H7 strains (ATCC 43888 and ATCC 700728).

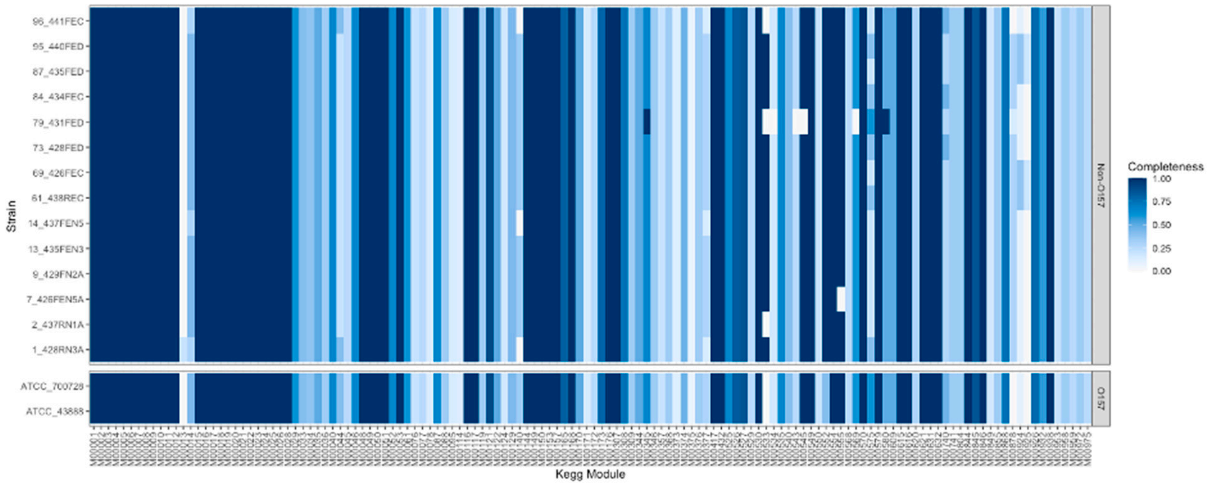

Supplementary Figure 6. Heatmap of KEGG module completeness in unique bovine non-O157:H7 *E. coli* strains and O157:H7 strains (ATCC 43888 and ATCC 700728). Blue denotes a gene as present and the intensity denotes the completeness of that module as determined from the Eggnog-mapper (v2.18) annotation of that strain genome. Pathway completeness was determined with ggKegg (v1.1.18).

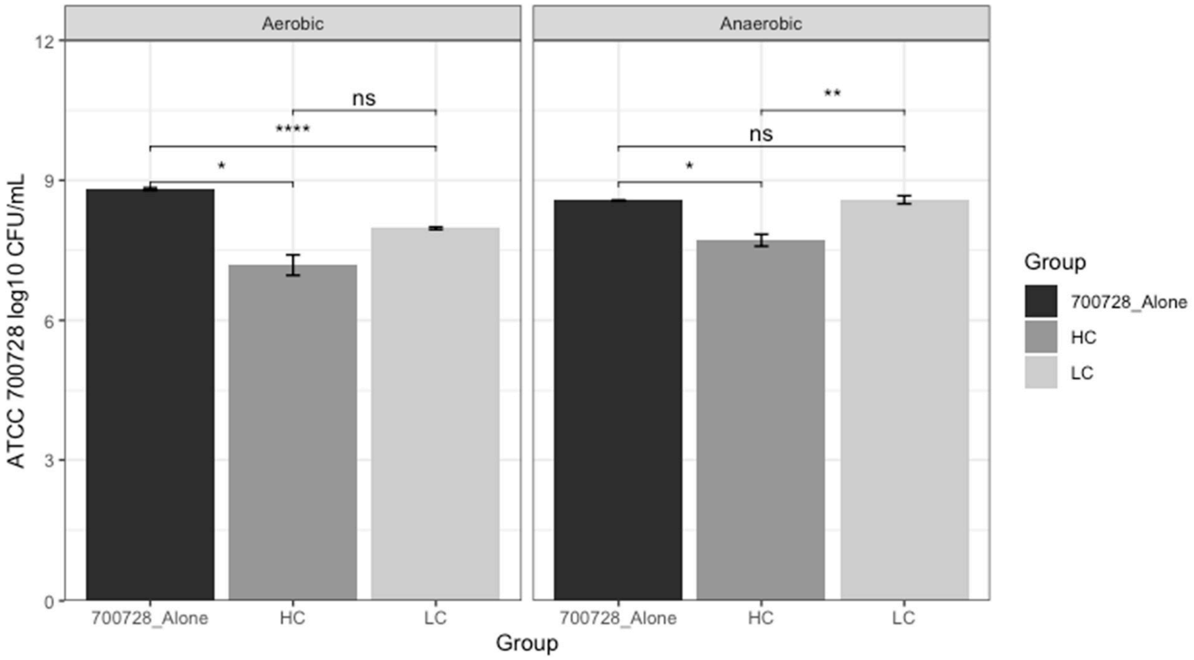

Supplementary Figure 7. Barcharts of *E. coli* O157:H7 ATCC 700728 counts (log_{10} CFU/mL) in competition assays with high competitive (HC) and low competitive (LC) consortia of unique bovine non-O157:H7 *E. coli* strains. *E. coli* O157:H7 ATCC 700728 counts were assessed after 24h of co-incubation with HC or LC consortia at 39ºC under either aerobic or anaerobic conditions. Counts were determined on Sorbitol MacConkey agar plates. Three biological replicates were conducted for each competition assay under each set of conditions. Colony forming units (CFU)/mL values were calculated, log_{10}-transformed, and statistically compared (t.test) in R using the dplyr (v1.1.4) and ggpubr (v0.6.0) packages. * indicates $p < 0.05$; ** indicates $p < 0.01$; *** indicates $p < 0.001$; "ns" indicates $p > 0.05$.

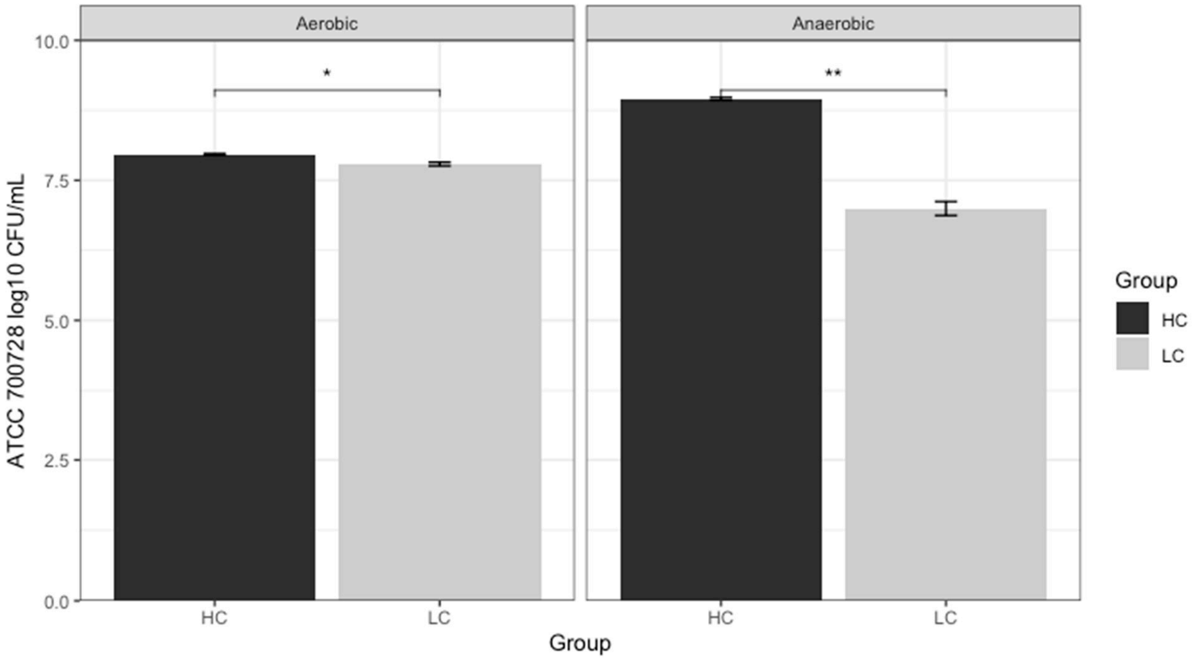

Supplementary Figure 8. Barcharts of bovine non-O157:H7 *E. coli* strain counts (log_{10} CFU/mL) in competition assays of high competitive (HC) and low competitive (LC) consortia of unique bovine non-O157:H7 strains against *E. coli* O157:H7 ATCC 700728. Bovine non-O157:H7 *E. coli* counts were assessed after 24h of co-incubation with *E. coli* O157:H7 ATCC 700728 at 39ºC under either aerobic or anaerobic conditions. Counts were determined on Sorbitol MacConkey agar plates. Three biological replicates were conducted for each competition assay under each set of conditions. Colony forming units (CFU)/mL values were calculated, log_{10}-transformed, and statistically compared (t.test) in R using the dplyr (v1.1.4) and ggpubr (v0.6.0) packages. * indicates $p < 0.05$; ** indicates $p < 0.01$; *** indicates $p < 0.001$; “ns” indicates $p > 0.05$.
